# A preoperative mathematic model for computed tomographic guided microwave ablation treatment of hepatic dome tumors

**DOI:** 10.18632/oncotarget.8299

**Published:** 2016-03-23

**Authors:** Fei Gao, Guo-Bao Wang, Zhan-Wang Xiang, Bin Yang, Jing-Bing Xue, Zhi-Qiang Mo, Zhi-Hui Zhong, Tao Zhang, Fu-Jun Zhang, Wei-Jun Fan

**Affiliations:** ^1^ Department of Interventional Radiology, Sun Yat-sen University Cancer Center, Guangzhou 510060, PR China; ^2^ State Key Laboratory of Oncology in South China, Guangzhou 510060, PR China; ^3^ Collaborative Innovation Center for Cancer Medicine, Guangzhou 510060, PR China; ^4^ Department of Endoscopy, Sun Yat-sen University Cancer Center, Guangzhou 510060, PR China; ^5^ Department of Gastrointestinal Surgery, Sun Yat-sen Memorial Hospital, Sun Yat-sen University, Guangzhou 510120, PR China; ^6^ Imaging Sciences, University of Rochester Medical Center, Rochester, NY 14642, USA

**Keywords:** CT-guided, microwave ablation, hepatic dome, tumors, mathematic model

## Abstract

**Purpose:**

This study sought to prospectively evaluate the feasibility and safety of a preoperative mathematic model for computed tomographic(CT) guided microwave(MW) ablation treatment of hepatic dome tumors.

**Methods:**

This mathematic model was a regular cylinder quantifying appropriate puncture routes from the bottom up. A total of 103 patients with hepatic dome tumors were enrolled and randomly divided into 2 groups based on whether this model was used or not: Group A (using the model; n = 43) versus Group B (not using the model; n = 60). All tumors were treated by CT-guided MW ablation and follow-up contrast CT were reviewed.

**Results:**

The average number of times for successful puncture, average ablation time, and incidence of right shoulder pain were less in Group A than Group B (1.4 vs. 2.5, *P* = 0.001; 8.8 vs. 11.1 minutes, *P* = 0.003; and 4.7% vs. 20%, *P* = 0.039). The technical success rate was higher in Group A than Group B (97.7% vs. 85.0%, *P* = 0.032). There were no significant differences between the two groups in primary and secondary technique efficacy rates (97.7% vs. 88.3%, *P* = 0.081; 90.0% vs. 72.7%, *P* = 0.314). No major complications occurred in both groups.

**Conclusion:**

The mathematic model of regular cylinder is feasible and safe for CT-guided MW ablation in treating hepatic dome tumors.

## INTRODUCTION

Image-guided microwave (MW) ablation, as an useful minimally invasive procedure, has been widely accepted for liver malignancies [1–5]). Although ultrasound (US) is the simplest and most popular modality for puncture guidance, the tumor sometimes locates in the hepatic dome and it is usually insufficiently visualized by sonography, which has a limited sonic window due to the overlapped lung or ribs. For such tumors, computed tomographic (CT) suggests an effective alternative for guidance and percutaneous themal ablation can be well achieved [6, 7].

However, MW ablation for hepatic dome tumors is usually technically challenging because of the potential risks of collateral thermal damage to the diaphragm. Thermal injury to the diaphragm can leads to pain, diaphragmatic paralysis, even rupture, perforation or fistula formation [8–12]. To reduce the risk of thermal injury to diaphragm and get a more sufficient ablation zone, most of operators select a puncture route from the bottom up since MW energy is emitted retrograde from the antenna tip [13, 14]. Nevertheless, CT does not allow for real-time surveillance of the puncture and a puncture route from the bottom up depends on the operator experience. Thus, we developed a mathematic model for puncture. A regular cylinder model, whose center of the top surface was also the center of tumor sphere, was established to quantify the appropriate puncture plane and route as a means to overcome the deficiencies that may be caused by lack of operator experience. So this study was to prospectively evaluate the feasibility and safety of this model for the quantification of CT-guided MW ablation in treating hepatic dome tumors.

## RESULTS

### Puncture and ablation parameters

All the patients were percutaneously treated in 124 CT-guided MW ablation sessions. The average number of punctures to ensure the correct antenna position in the targeted tumor was less in Group A than Group B (1.4 ± 0.7 [range, 1-3 punctures] vs. 2.5 ± 1.2 [range, 1-6 punctures], *P* = 0.001). The average time of ablation was less in Group A than Group B (8.8 minutes ± 2.8 [range, 5-15 minutes] vs. 11.1 minutes ± 4.2 [range, 5-20 minutes], *P* = 0.003). In Group A, the mean puncture angle between the seleted vertical plane and the coronal plane was 48.6° ± 29.0 (range, 0-90°). The mean puncture angle in the vertical plane was 44.8° ± 10.8 (range, 32-65°). The mean puncture length was 15.5 cm ± 3.3 (range, 8.5-20.0 cm).

### Technique efficacy

#### Technical success

The technical success rate (97.7%, 42/43) in Group A was significantly higher than that of Group B (85.0%, 51/60) for the initial session (*P* = 0.032). In Group A, 1 tumor (2.3% [1/43]), which were probably irregularly shaped and not consistent with the results of the mathematic calculations, was not covered completely by the ablation zone after overlapping ablation. In Group B, 9 tumors (15.0% [9/60]) were not covered completely by the ablation zone after overlapping ablation.

#### Primary and secondary technique efficacy rate

At the one month follow-up CT, the primary technique efficacy rate was 97.7% (42/43) for Group A, compared to 88.3% (53/60) for Group B (P = 0.081). The secondary technique efficacy rates were evaluated in 21 patients who had a 1 year follow-up with CT. It was 90.0% (9/10) in Group A, compared to 72.7% (8/11) in the Group B (P = 0.314).

### Local control rate and local tumor progression rate

The local control rate and the local tumor progression rate were listed in Table 2. For the patients of Group A, the local control rate of 3, 6, 12, 24, 36 months was 88.4%, 86.0%, 79.1%, 62.8%, 55.8% respectively, and the local tumor progression rate was 4.7%, 7.0%, 11.6%, 23.3%, 39.5% respectively. In Group B, the local control rate of 3, 6, 12, 24, 36 months was 91.7%, 86.7%, 76.7%, 56.9%, 46.6% respectively, and the local tumor progression rate was 3.3%, 5.0%, 15.0%, 37.9%, 51.7% respectively. The local control rate and local tumor progression rate of 3, 6, 12, 24, 36 months had no significant differences between the two groups (*P =* 0.578, 0.928, 0.773, 0.551, 0.357 respectively; and *P =* 0.733, 0.673, 0.622, 0.117, 0.225 respectively).

**Table 1:** Patient characteristics summary

Characteristic	Group A (n=43)	Group B (n=60)	*P*
Age (years)			
Mean±SD	59.3±10.3 (37-75)	61.2±9.2 (41-77)	0.335
≤60	22	25	0.340
>60	21	35	
Sex			0.773
Male	34	46	
Female	9	14	
Tumor diameter (cm)			
Mean±SD	1.8±0.8(0.5-3.4)	1.9±0.8(0.5-3.6)	0.763
≤1	8	12	0.872
>1, ≤2	23	29	
>2	12	19	
Primary tumor			0.828
HCC	21	28	
CLM	22	32	
ECOG PS*			0.793
0	32	41	
1	10	17	
2	1	2	

Note.—Group A: CT-guided MW ablation using the mathematic model, Group B: CT-guided MW ablation not using the mathematic model; HCC: Hepatocellular carcinoma; CLM: Colorectal liver metastasis;

* ECOG PS: Eastern Cooperative Oncology Group performance status.

**Table 2:** Responses of patients for CT-guided MW Ablation of hepatic dome tumors

Group/Time (months)	Number of patients	CR	PR	SD	PD	Local control rate (%)	Local tumor progression rate (%)
Group A							
3	43	33	5	3	2	88.4(38/43)	4.7(2/43)
6	43	31	6	3	3	86.0(37/43)	7.0(3/43)
12	43	28	6	4	5	79.1(34/43)	11.6(5/43)
24	39	25	2	6	6	62.8(27/43)	23.3(10/43)
36	31	23	1	3	5	55.8(24/43)	39.5(17/43)
Group B							
3	60	49	6	3	2	91.7(55/60)	3.3(2/60)
6	60	45	7	5	3	86.7(52/60)	5.0(3/60)
12	57	38	8	5	6	76.7(46/60)	15.0(9/60)
24	41*	31	2	3	5	56.9(33/58)	37.9(22/58)
36	32*	26	1	1	4	46.6(27/58)	51.7(30/58)

Note.—Group A: CT-guided MW ablation using the mathematic model, Group B: CT-guided MW ablation not using the mathematic model; CR: Complete response, PR: Partial response, SD: Stable disease, PD: Progressive disease;

* Two patients in group B who were alive without evidence of recurrence at 15, 20 months at the time of writing were excluded at 24 and 36 months of follow-up time.

### Overall survival (OS) and tumor-free survival (TFS)

During the follow-up, the estimated mean OS was 33.3 months for Group A, which had no significant difference with that of Group B ( 29.8 months, *P* = 0.062; Figure 4). The estimated mean TFS was 23.7 months for Group A, which had also no significant difference with that of Group B (22.1 months, *P* = 0.501).

### Complications

There were no deaths related to all the MW ablation procedures. Several minor complications or side effects related to MW ablation occurred during or after the procedure (Table 3). A small amount of hepatic sub-capsular hematoma was found in 5 patients which was automatically absorbed by the body (less than 50ml by the estimation on CT scanning; 1 case in Group A and 4 cases in Group B). A small sized pulmonary hematoma occurred in another 4 patients that involved antenna insertion through the lung, which was also automatically absorbed (1 cases in Group A and 3 cases in Group B). None of these patients with hepatic sub-capsular or pulmonary hematoma had symptoms and received only conservative treatment without transfusion. Five patients presented with a pneumothorax with pulmonary compression of 10-40% during the procedure (1 case in Group A and 4 cases in Group B). However, these patients had no respiratory symptoms. Only one of these patients in Group B needed drainage. During the follow-up, mild right shoulder pain developed in 14 patients and lasted for 1-3 weeks after the MW ablation (2 cases in Group A and 12 cases in Group B). The symptoms were resolved with conservative treatment. The incidences of complications or side effects between the two groups had no significant differences except right shoulder pain. Major complications such as massive bleeding, diaphragmatic rupture, perforation, or fistula formation did not occur in either group.

**Table 3:** Incidence rates of complications following MW ablation between Group A and Group B

Complications	Group A (%, n = 43)	Group B (%, n = 60)	*P*
*Major complication*	0	0	
*Minor complications and side effects*			
A small amount of hepatic sub-capsular hematoma	2.3 (1/43)	6.7 (4/60)	0.397
A small sized pulmonary hematoma	2.3 (1/43)	5.0 (3/60)	0.638
Pneumothorax	2.3 (1/43)	6.7 (4/60)	0.397
Right shoulder pain	4.7 (2/43)	20.0 (12/60)	**0.039**

Note.—Group A: CT-guided MW ablation using the mathematic model; Group B: CT-guided MW ablation not using the mathematic model

## DISCUSSION

There's no study which has been performed to determine a mathematic model for CT-guided ablation of hepatic dome tumors. These tumors are usually covered by the diaphragm and a small amount of normal liver tissues. As a result, there's a limited space available for MW antenna placement and it's difficult to enlarge the ablation zone using a puncture route in the axial plane. So in this study, each puncture route was designed from the bottom up to reduce the potential risks of diaphragmatic thermal damage because of retrograde emission of MW energy from the antenna tip [13, 14]. By using this model, a puncture route from the bottom up can be well quantified for antenna placement. Both the average number of times for successful puncture and the average time of ablation were reduced in the study group. The antennas are easier to be controlled since a relatively accurate puncture can be achieved by a quantified puncture route. Although there's no survival advantage by using this model, it has overcomed the limitation of puncture mainly by operator experience to some extent since CT can only provide an intermittent guidance.

We have also noticed some reports about US-guided thermal ablation with artificial fluid for hepatic dome tumors [15–22]. The artificial fluid is a good method to improve the sonic window and separate the adjacent diaphragm from the tumor. Nevertheless, this technique is relatively complicated and may not be suitable for junior operators. Also, the cold artificial fluid may lower the temperature of its surrounding, which could be an obstacle to a successful thermal ablation. On the contrary, CT facilitates detection of hepatic dome tumors without any infusion of artificial fluid and the diaphragm surrounding the tumor can be clearly displayed [6, 7, 23]. With this model, we also got satisfied outcomes in treating such challenging cases and the technique efficacy was similar to some prior reports [24, 25]. Although the technical success rate was higher for the study group, the primary and secondary technique efficacy between the two groups had no significant difference. It can be explained that the average ablation time of the control group was significantly longer than the study group.

In our study, right shoulder pain after ablation can be explained by the adhesion between coagulative necrotic tissues and adjacent diaphragm caused by ablation. It is regarded as a signal of thermal transmission exactly to the diaphragm or mild diaphragmatic thermal damage [24–26]. The incidence of right shoulder pain was significantly lower for the study group, suggesting less diaphragmatic thermal damage occurred with this model. We consider that it was associated with less of ablation time by the model. We also expected that thermal damage of the diaphragm would have been less likely with a power of 45 W for 5-10 minutes per application. This was according to the proposed guidelines for diameters based on different time and power variables, which recommended a power of 45 W for 10 minutes to achieve optimal diameters at the shortest time and lowest wattage [27, 28]. Since a small amount of hepatic sub-capsular hematoma was found in both groups, we paid close attention to the affection by breathing during puncture to reduce the risk of lacerations of the liver capsule, which could cause massive bleeding [29, 30]. All the punctures were performed at the end of expiration in this study to reduce the effects of breathing. The occurrence of pneumothorax and a small sized pulmonary hematoma was due to the insertion of the antenna through the lung since the transpulmonary routes were planned in some of the patients. Even if these complications occurred, they would not cause serious problems for localization and ablation of the intrahepatic tumors. No major complications occurred, suggesting CT-guided MW ablation with this mathematic puncture model is a safe procedure.

For the application of this model, it is important to confirm whether the antenna crosses the marked center of tumor's maximal circle in the axial plane. This is to ensure the overlap of the ablation sphere's center and the tumor sphere's center, which could lead to more complete ablation of tumor according to our model. For some tumors with relatively irregular shapes, overlapping ablation based on the CT scan is needed when the necrotic zone does not completely cover the tumor only by increasing ablation time. Based on our experience, the puncture site is usually selected 2-3 intercostal spaces below the axial plane which has the tumor's maximal area. Although a lower puncture site theoretically leads to lower risks of diaphragmatic thermal damage, more than 3 intercostal spaces is not recommended due to the added puncture length and increased difficulty in successful puncture. A coronal plane is recommended as the described vertical plane for puncture in the cylinder model because of better controllability of antennas.

The described model was limited by the fact that the tumor and ablation zone were not always spherical. Thus, CT scan during the procedure and the comparison with established model were valuable. It is necessary to thoroughly understand the three-dimensional features of tumors before procedure. Our study also had limitations that the tumors enrolled were not larger than 3.6 cm and we only followed the patients up for a maximum of 36 months. Larger tumors and a longer follow-up are desired for future study.

In conclusion, we developed a feasible and safe mathematic model for CT-guided MW ablation in treating hepatic dome tumors, which might be an alternative mode to quantify puncture routes and ablate hepatic dome lesions.

## PATIENTS AND METHODS

### Ethics

This prospective study has been carried out in accordance with the Declaration of Helsinki (2000) of the World Medical Association. It was approved by Sun Yat-sen University Cancer Center Institutional Review Board. All patients signed informed written consent.

### Patients

From May 2008 to April 2013, 103 patients (80 men and 23 women; mean age: 60.4 years ± 9.7 [SD], range: 37-77 years) with a total of 103 tumors located in the hepatic dome, including 49 cases of hepatocellular carcinoma (HCC) and 54 of colorectal liver metastasis (CLM), were enrolled in this study for CT-guided MW ablation. The enrolled patients were randomly divided into 2 groups based on whether a preoperative mathematic puncture model was used or not before ablation: Group A (using the model; n = 43) versus Group B (not using the model; n = 60). Randomization was achieved by means of computer-generated random numbers. The mean size of the tumor was 1.8 cm ± 0.8 (range, 0.5-3.6 cm) in the largest diameter. Of the 49 patients with HCC, 32 (65.3%) had stage I or II tumors (TNM system); 17 (34.7%) had hepatic dome recurrence after surgical resection, MW ablation, radiofrequincy (RF) ablation or transcatheter arterial chemoembolization (TACE). Of the 54 patients with CLM, treatment of the primary cancer were all surgical resection. There were no significant differences between patient characteristics of Group A and Group B (Table 1).

Our diagnosis was confirmed with pathologic proof at CT-guided needle biopsy before MW ablation in all patients. All the enrolled patients met the following inclusion criteria: (a) A single tumor located in the hepatic dome and not well detectable by US. (b) For the cases of recurrent HCC and CLM, the hepatic dome tumor was the only evidence of recurrence. (c) Eastern Cooperative Oncology Group performance status (ECOG PS) ≤ 2. (d) Child-Pugh class A or class B (total serum bilirubin < 2.0 mg/dl ). (e) The patients refused surgical resection for hepatic dome tumors. (f) Adequate renal function (serum creatinine < 2.0 mg/dl). (g) Leucocyte count > 3.0 × 10^9^/L, platelet count > 50.0 × 10^9^/L. Patients were excluded if they met any of the following criteria: (a) There was related malignant pleural effusion. (b) Serious comorbidities.

### Preoperative tumor targeting

#### Group A: mathematic model establishment and puncture route calculation

**Figure 1:**
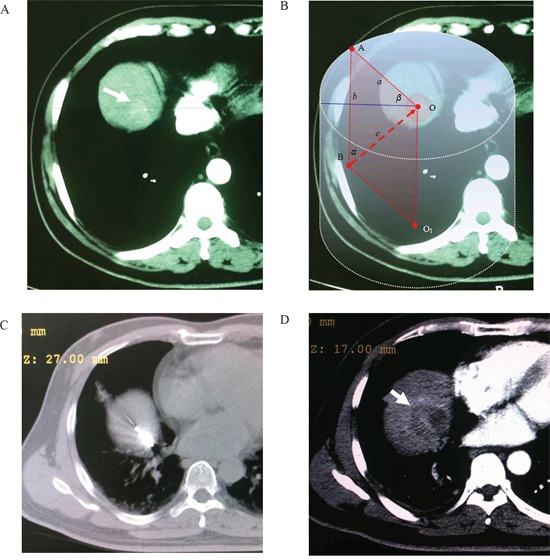
Application of the mathematic puncture model Images obtained in a 55-year-old man with HCC. **A.** Preoperative transverse CT scan showed An enhanced lesion located in the hepatic dome (arrowhead). **B.** The maximal area of tumor in the axial plane was selected and a circular delineation was performed. A cylinder model was established after selecting the center of tumor's maximal circle in axial plane as its top surface center (point “O”). A vertical plane which crossed point “O” and “A” (the skin poin on the circle of cylinder's top surface) was determined (β = 45°). According to the calculation method of this model, the puncture route was determined as “BO” after selection of puncture site “B”. **C.** A MW antenna was percutaneously inserted into the lesion from the bottom up along the puncture route caculated by the established model (α = 45°, *d* = 11.3 cm). **D.** Arrow indicated complete necrosis of tumor without enhancement after ablation.

**Figure 2:**
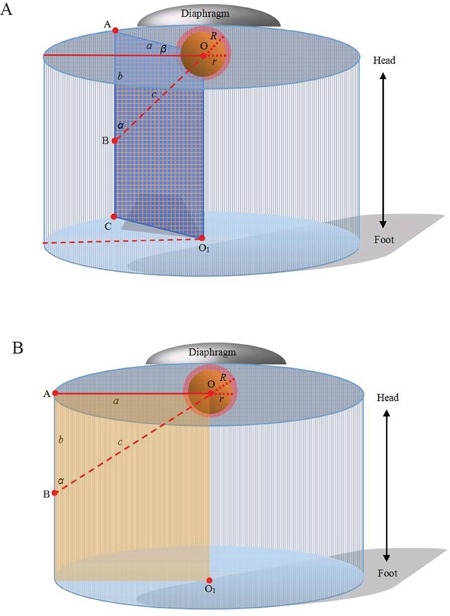
Computer representations of regular cylinder model **A.** Orthostatic transillumination view of the general model: A tumor sphere's center is the same as the center of a cylinder's top surface (point “O”). The maximal circle of the tumor sphere in axial plane also locates in the cylinder's top surface. A puncture site locats in the axial plane 2-3 intercostal spaces below the cylinder's top surface (point “B”). Then a regular cylinder model whose center of the top surface was point “O” was established crossing point “B” on its side surface. A plane inside the cylinder which crosses point “O” and “B” is vertical to the top surface (also passing through the center of the cylinder's bottom surface, point “O_1_”). The projection of the puncture site on the cylinder's top surface (point “A”), point “B” and “O”, form a right triangle in the vertical plane. The length of “OB” (“*c*”) can be calculated by the measurement of “OA” (“*a*”) and “AB” (“*b*”). The hypotenuse of the right triangle is the puncture route. The puncture angle between the vertical plane and the coronal plane can be measured (“*β*”) and the angle in the vertical plane can be calculated by the measurements of “*a*” and “*b*” in the right triangle. The puncture length (“*d*”) is the hypotenuse length plus ablation radius (“*R*”) which consists of the tumor radius (“*r*”) plus at least a 0.5-cm tumor-free margin. So, $d=c+R=\sqrt{a^{2}+\;b^{2}}+R;\alpha =\text{arctg}\frac{a}{b}$. **B.** Orthostatic transillumination view of a special model: When *β* is 0, the vertical plane is overlapped by the coronal plane which is passing through point “O”. In our experience, this special model is more simple for puncture and the coronal plane is easier to control antennas.

Before the mathematic model of a regular cylinder was established, we assumed that both the tumor and ablation zone were perfect spheres with the same spheric center. The mathematic analysis was performed to determine how an antenna could cross the center of the tumor sphere from the bottom up for an efficient ablation. According to the assumption above, the center point of the tumor sphere's maximal circle in the axial plane was also the tumor sphere's center point. CT images (Siemens Brilliance Big Bore, Germany; 0.625 mm collimation beam at 200 mA and 120 kVp) with 2-5 mm section thickness were obtained in expiration for targeting tumor before MW ablation (Figure 1A, 3A). The maximal area of the tumor in the axial plane was selected and a careful circular delineation was performed. This enabled determining and marking the center point of the tumor's maximal circle in axial plane, which was also the center point of the tumor sphere and cylinder's top surface. A puncture site, which located in the axial plane 2-3 intercostal spaces below the cylinder's top surface, was selected. Then a regular cylinder model whose center of the top surface was also the tumor sphere's center was established crossing the selected puncture site on its side surface (Figure 1B). Inside the cylinder, a vertical plane, which was vertical to the cylinder's top surface, was confirmed by the puncture site and the tumor sphere's center point. After selecting the projection of the puncture site on the cylinder's top surface, a right triangle in the vertical plane was confirmed by three points: the center of tumor sphere, the puncture site and its projection. The hypotenuse was the puncture route from the bottom up which was used to ablate hepatic dome tumors. The puncture length was the hypotenuse length plus ablation radius which consisted of the tumor radius plus at least a 0.5-cm tumor-free margin. The puncture angle between the seleted vertical plane and the coronal plane was directly achieved by CT measurement. The angle in the vertical plane was calculated by the two legs of the right triangle measured by CT. The schematic of the mathematic model is demonstrated in Figure 2.

**Figure 3:**
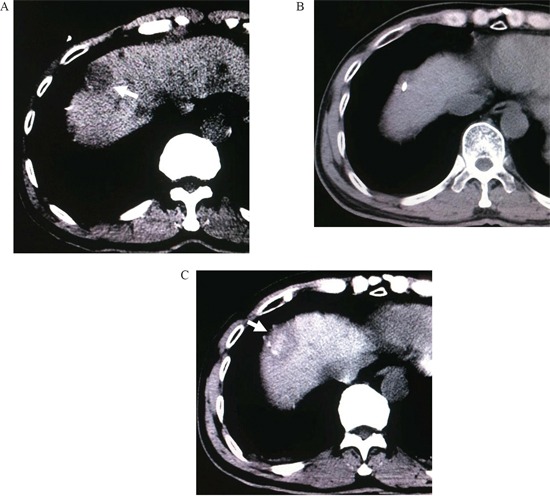
**A.** CT scan before treatment, a hepatic dome lesion can be found (arrowhead). **B.** A MW antenna was inserted into the lesion by the established model. **C.** Complete necrosis of tumor without enhancement after ablation (arrowhead).

**Figure 4:**
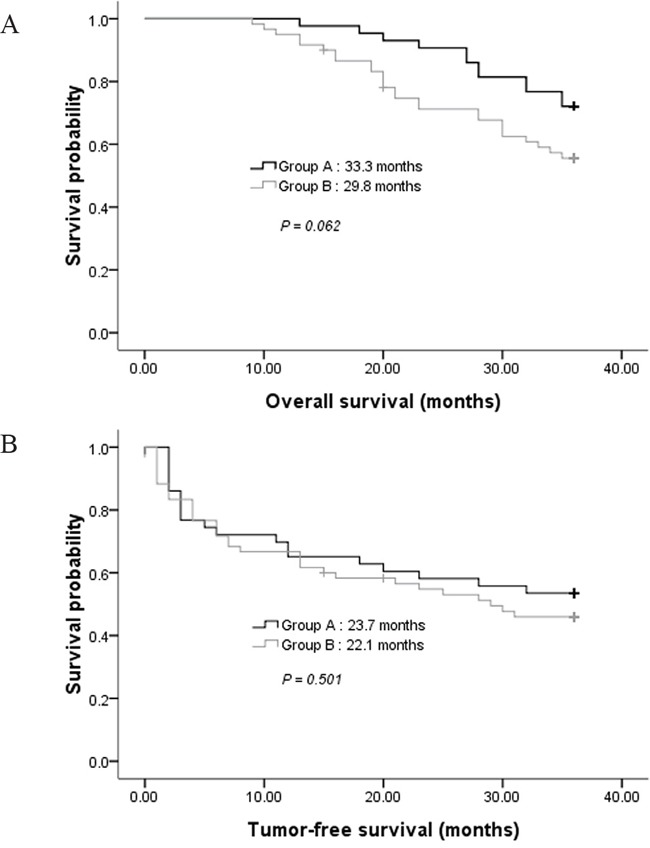
**A.** Comparison of overall survival in groups A and B. **B.** Comparison of tumor-free survival in groups A and B.

#### Group B

The mathematic model above was not used for the patients of Group B. After CT images (the same equipment and scanning parameters as Group A) with 2-5 mm section thickness were obtained for targeting tumor before MW ablation, the center point of the tumor's maximal circle in axial plane was marked. Then a puncture site, which located in the axial plane 2-3 intercostal spaces below the axial plane of the tumor's maximal circle was selected. A puncture route was planned to cross the center point of the tumor's maximal circle from the bottom up.

### Ablation procedure

#### Puncture

All the punctures were performed by the same two interventional radiologists (F.G., F.J.Z.), each of whom had more than 10 years of experience in CT-guided MW ablation. The patients were in the supine position on CT table and all the punctures were performed in expiration. After local infiltration anesthesia was induced by using 5-15 mL of 1% lidocaine (Liduokayin; Yimin, Yichang, China), a antenna (water-cooled, diameter 1.7 mm; made by Qi Ya Medical Treatment Facility Limited Company, Nanjing, China) was percutaneously inserted into the targeted tumor. In order to reduce the effects of breathing, all the punctures were performed at the end of expiration in this study. In Group A, the puncture was performed via the selected puncture site along the puncture route caculated by the model under CT guidance (Figure 1C, 3B). In Group B, the puncture was performed only under CT guidance. The puncture angles in Group A were according to a simple protractor whose angle was caculated by the model.

#### Verification of the puncture route

In both groups, axial CT scan was obtained to confirm whether the antenna passed through the marked center of tumor's maximal circle in axial plane after puncture. Also, it had to be confirmed that the antenna tip had reached the superior most axial CT slice that contained tumor. The antenna was adjusted slightly until it crossed that center and reached the top slice of the tumor. The interval between the tip and diaphragmatic muscle should be more than 5 mm.

#### Ablation

In both groups, general anesthesia was induced with intravenous administation of propofol (Diprivan; Zeneca, Macclesfield, United Kingdom) (1-2 mg/kg) and fentanyl (Fentaini; Renfu, Yichang, China) (50-100 μg) after puncture. The energy of MW ablation was applied with a power of 45 W for 5-10 minutes per MW application. The time of MW ablation were determined on the basis of lesion necrosis observed on the CT scan during the procedure (Emission facility: frequency, 2450 MHz; output power, 0-120 W; precision of temperature control: ±0.1°C; discharge waveform: continuous wave; produced by Qi Ya Medical Treatment Facility Limited Company, Nanjing, China) (Figure 1D, 3C).

#### Overlapping ablation

In both groups, overlapping ablation was needed when the shape of tumor was relatively irregular and the necrotic zone did not completely cover the tumor only by increasing ablation time. It was achieved after slight position adjustment of the antenna based on CT scanning during the procedure.

### Follow-up

According to our follow-up protocol, images of abdominal contrast CT were obtained immediately, 1, 3 months after MW ablation, and then at 3-month intervals. The therapeutic efficacy, including the technical success rate, primary technique efficacy rate and secondary technique efficacy rate were evaluated. The technical success addressed whether the tumor was treated according to protocol and was covered completely by the ablation zone. Specifically, the technical success rate was assessed with contrast-enhanced CT immediately following the procedure. The primary technique efficacy rate was defined as the percentage of tumors that were successfully eradicated at the one month follow-up CT. The secondary technique efficacy rate was determined by a 1-year and included cases that underwent successful repeated percutaneous MW ablations following the identification of local tumor progression.

We also evaluated the response according to the Response Evaluation Criteria in Solid Tumors (RECIST). Specifically, it was assessed by the presence of contrast enhancement in the ablation area. Lack of enhancement was deemed to indicate complete ablation and no evidence of local progression, while residual enhancement or appearance of foci of enhancement in the ablation area was deemed to indicate incomplete ablation or local progression, respectively. Local control rate was defined as the proportion of patients who received complete response (CR) and partial response (PR). Local tumor progression was diagnosed with the identification of untreated disease foci in tumors that were previously considered to be completely ablated. These tumors had to be considered completely ablated by the definition used for primary technique efficacy. All the follow-up CT scans were evaluated by the consensus of two radiologists (F.G., F.J.Z.).

A helical scanner (HiSpeed Advantage, GE Healthcare, Milwaukee, WI) was used with a 5-mm slice thickness as in the pre-procedural imaging study. A total of 120 mL of nonionic contrast material (Ultravist 300 [iopromide, 300 mg I/mL], Schering, Berlin, Germany) was administered at a rate of 3 mL/sec with an automatic power injector. The scan images were acquired at pre-injection, 30, 70, and 180 sec after IV contrast material injection, which represented the non-enhanced, hepatic arterial, portal venous, and equilibrium phases, respectively. All scans were obtained by using a 0.625 mm collimation beam at 200 mA and 120 kVp.

### Statistical analyses

The characteristics of the patients in the two groups were compared by independent *t* test and Pearson's *χ*^2^ test. The average number of punctures to ensure the correct antenna position in the targeted tumor, and the average time of ablation were compared by independent *t* test. A Pearson's *χ*^2^ test and Fisher's exact test were used to analyze differences in the complication rate and therapeutic efficacy. Survival was calculated by Kaplan-Meier analysis and statistical significance was determined by Mantel-Cox log rank test. The statistical analyses were performed by using SPSS 17.0 statistical software (SPSS, Chicago, III). All statistical tests were two sided and *P* < 0.05 was considered significant.
